# Impact of extracellular matrix on engraftment and maturation of pluripotent stem cell-derived cardiomyocytes in a rat myocardial infarct model

**DOI:** 10.1038/s41598-017-09217-x

**Published:** 2017-08-17

**Authors:** Tatsuki Ogasawara, Satomi Okano, Hajime Ichimura, Shin Kadota, Yuki Tanaka, Itsunari Minami, Motonari Uesugi, Yuko Wada, Naoto Saito, Kenji Okada, Koichiro Kuwahara, Yuji Shiba

**Affiliations:** 1 Institute for Biomedical Sciences, Shinshu University, Matsumoto, Japan; 2 Department of Regenerative Science and Medicine, Shinshu University, Matsumoto, Japan; 3 Department of Cardiovascular Surgery, Shinshu University, Matsumoto, Japan; 4 Department of Cardiovascular Medicine, Shinshu University, Matsumoto, Japan; 5 Department of Cell Design for Tissue Construction, Osaka University, Suita, Japan; 6 Institute for Integrated Cell-Material Sciences (WPI-iCeMS) and Institute for Chemical Research, Kyoto University, Uji, Japan

## Abstract

Pluripotent stem cell-derived cardiomyocytes show great promise in regenerating the heart after myocardial infarction; however, several uncertainties exist that must be addressed before clinical trials. One practical issue is graft survival following transplantation. Although a pro-survival cocktail with Matrigel has been shown to enhance graft survival, the use of Matrigel may not be clinically feasible. The purpose of this study was to test whether a hyaluronan-based hydrogel, HyStem, could be a substitute for Matrigel. Human induced pluripotent stem cell-derived cardiomyocytes diluted with HyStem alone, HyStem plus pro-survival factors, or a pro-survival cocktail with Matrigel (PSC/MG), were transplanted into a rat model of acute myocardial infarction. Histological analysis at 4 weeks post transplantation revealed that, among the three groups, recipients of PSC/MG showed the largest graft size. Additionally, the grafted cardiomyocytes in the recipients of PSC/MG had a more matured phenotype compared to those in the other two groups. These findings suggest that further studies will be required to enhance not only graft size, but also the maturation of grafted cardiomyocytes.

## Introduction

Despite remarkable progress in pharmacological and recanalization therapies during the last several decades, ischemic heart disease remains the major cause of death worldwide^1^. Current therapies can ameliorate the progress of heart failure following a heart attack but cannot reverse the loss of myocardium function, as adult cardiomyocytes have a limited capacity for proliferation^2^. Pluripotent stem cells have been extensively investigated for cardiac repair, and a substantial amount of proof-of-concept evidence has been established^3–7^. However, several uncertainties still remain regarding the use of pluripotent stem cell-derived cardiomyocytes, which must be addressed before clinical application. A remaining practical question concerns whether enough cardiomyocytes can be generated at an affordable cost. On average, a billion cardiomyocytes are lost after a typical myocardial infarction^8^. Given that many pluripotent stem cell-derived cardiomyocytes die after transplantation into the ischemic myocardium^9^, at least several billion cardiomyocytes are required. Additional research with a focus on reducing the cost is needed, including the development of more efficient cardiomyocyte generation and regulatory validation of the cell products. Another approach that would reduce the cost is to enhance graft survival following cell transplantation. Our group, as well as others, have reported that the use of a pro-survival cocktail (PSC) results in significantly larger surviving grafts^3, 10^. PSC consists of caspase inhibitor ZVAD, Bcl-X_L_ BH4, cyclosporine, IGF-1, pinacidil, and Matrigel, which synergistically enhance graft survival. Matrigel is a chemically undefined protein mixture, derived from a mouse sarcoma cell line. It is doubtful that the use of Matrigel is clinically feasible and safe, suggesting the need for a substitute. Hyaluronan is a glycosaminoglycan component of the extracellular matrix found in all connective tissues, and a hyaluronan-based hydrogel, HyStem-C, has been shown to enhance the survival of cardiosphere-derived cells transplanted into infarcted mouse hearts^11^. In the present study, we tested whether hyaluronan-based hydrogel enhances graft survival of induced pluripotent stem (iPS) cell-derived cardiomyocytes and could serve as a substitute for Matrigel.

## Results

### Partial remuscularization of the infarcted myocardium by human iPS cell-derived cardiomyocytes

**Figure 1:**
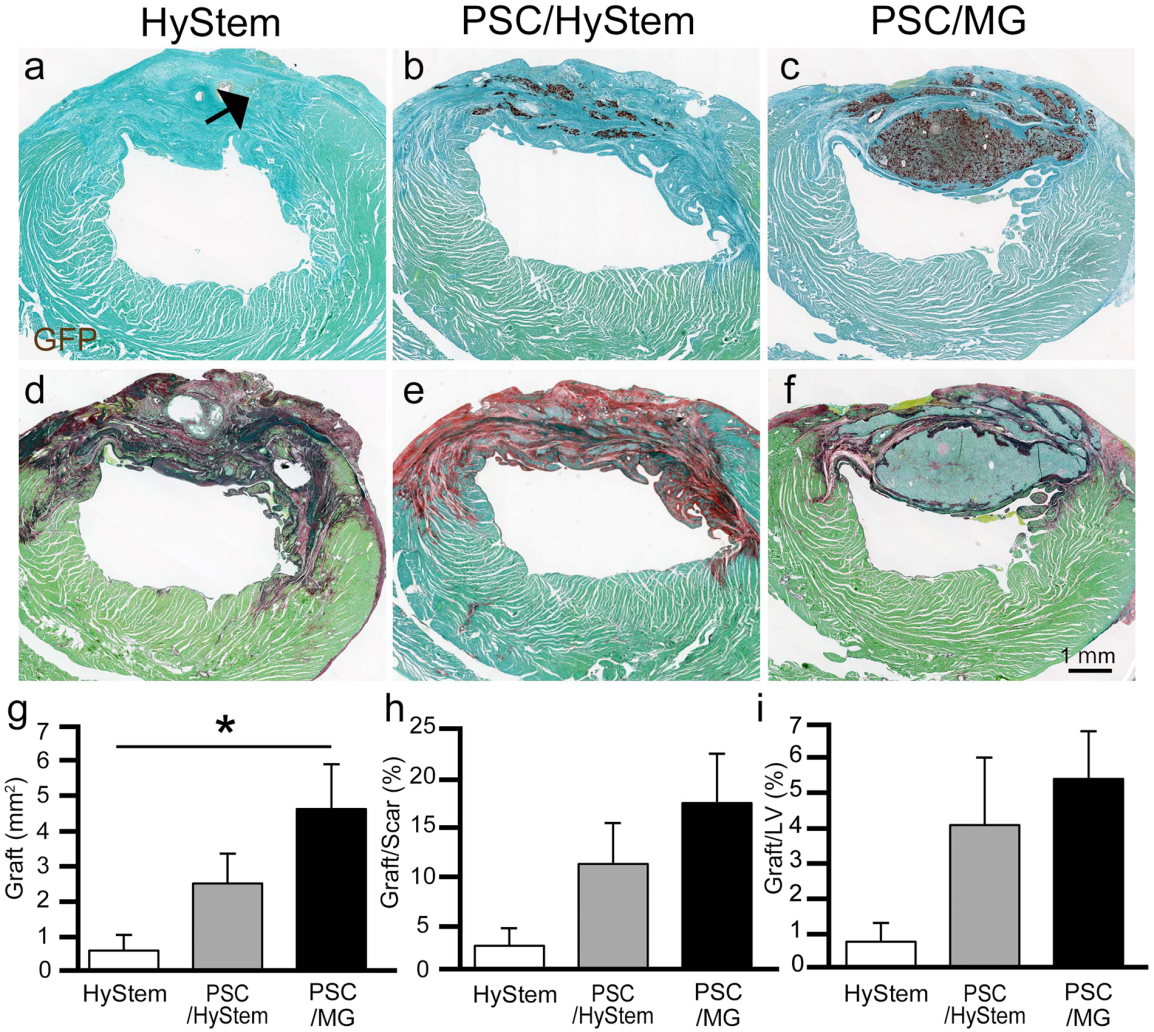
Partial remuscularization of the infarcted myocardium using human iPS cell-derived cardiomyocytes is shown. A week after myocardial infarction induction, human iPS cell-derived cardiomyocytes, diluted with HyStem (n = 5), PSC/HyStem (n = 5), or PSC/MG (n = 7), were transplanted directly into the infarcted myocardium. The heart was excised for histological analysis at 4 weeks post transplantation. (**a**–**c**) Immunostaining against the graft marker with GFP (brown DAB deposit), counterstained with fast green. While only a small portion of grafted tissue (arrow) was observed in the HyStem group, a substantial amount of grafted tissue was observed in the PSC/HyStem and PSC/MG groups. (**d**–**f**) Picro-sirius red staining of sections in close proximity to (**a**–**c**) show that most of the grafted tissue was located in the infarcted scar tissue. (**g**) Graft area, *P < 0.05. (**h**) Graft area relative to scar area. (**i**) Graft area relative to the left ventricular area. PSC, pro-survival cocktail; MG, Matrigel; GFP, green fluorescent protein; DAB, diaminobenzidine.

Cardiomyocytes were generated from our previously reported GCaMP3-expressing^4^ 253G1 iPS cell line using a modified version of a protocol previously developed by our group^12^. Flow cytometry was used to assess cardiomyocyte purity; the transplanted iPS cell-derived cardiomyocytes were 86.8% ± 2.5% positive for cardiac troponin T (Supplementary Fig. 1). All of the cardiomyocytes were heat-shocked^9^ and cryopreserved for subsequent transplantation studies. The myocardial infarction model was produced by ligation of the left anterior descending artery in T cell-deficient rats. One week after myocardial infarction induction, 2 × 10^7^ iPS cell-derived cardiomyocytes, diluted with either HyStem-C alone (HyStem; n = 5), a pro-survival cocktail in which Matrigel was replaced with HyStem-C (PSC/HyStem; n = 5), or a pro-survival cocktail with Matrigel (PSC/MG; n = 7), were injected directly into the myocardium (Supplementary Fig. 2). Histological analysis at 4 weeks post transplantation showed partial remuscularization of the infarcted scar tissue with graft cells in all three groups (Fig. 1a–i). The animals in the PSC/MG group had the largest graft area (HyStem, 0.6 ± 0.4 mm^2^; PSC/HyStem, 2.5 ± 0.8 mm^2^; PSC/MG, 4.6 ± 1.2 mm^2^; P < 0.05 HyStem vs. PSC/MG; Fig. 1g), resulting in the remuscularization of 16.7% ± 4.9% of the scar tissue (Fig. 1c,f,h). Almost all graft cells had cardiac troponin T (cTnT)-positive cardiomyocytes (% of cTnT: HyStem, 100%; PSC/HyStem, 99.7% ± 0.3%; PSC/MG, 97.8% ± 1.2% of the graft area, Supplementary Fig. 3a,b). However, there was only a small portion of cartilage in the grafts (PSC/HyStem, 1 out of 5 animals, 0.3% ± 0.3% of graft area; PSC/MG, 3 out of 7 animals, 2.1% ± 1.2% of the graft area, Supplementary Fig. 3c,d).

### Recipients of iPSC-CMs in PSC/MG had the largest graft cardiomyocytes

**Figure 2:**
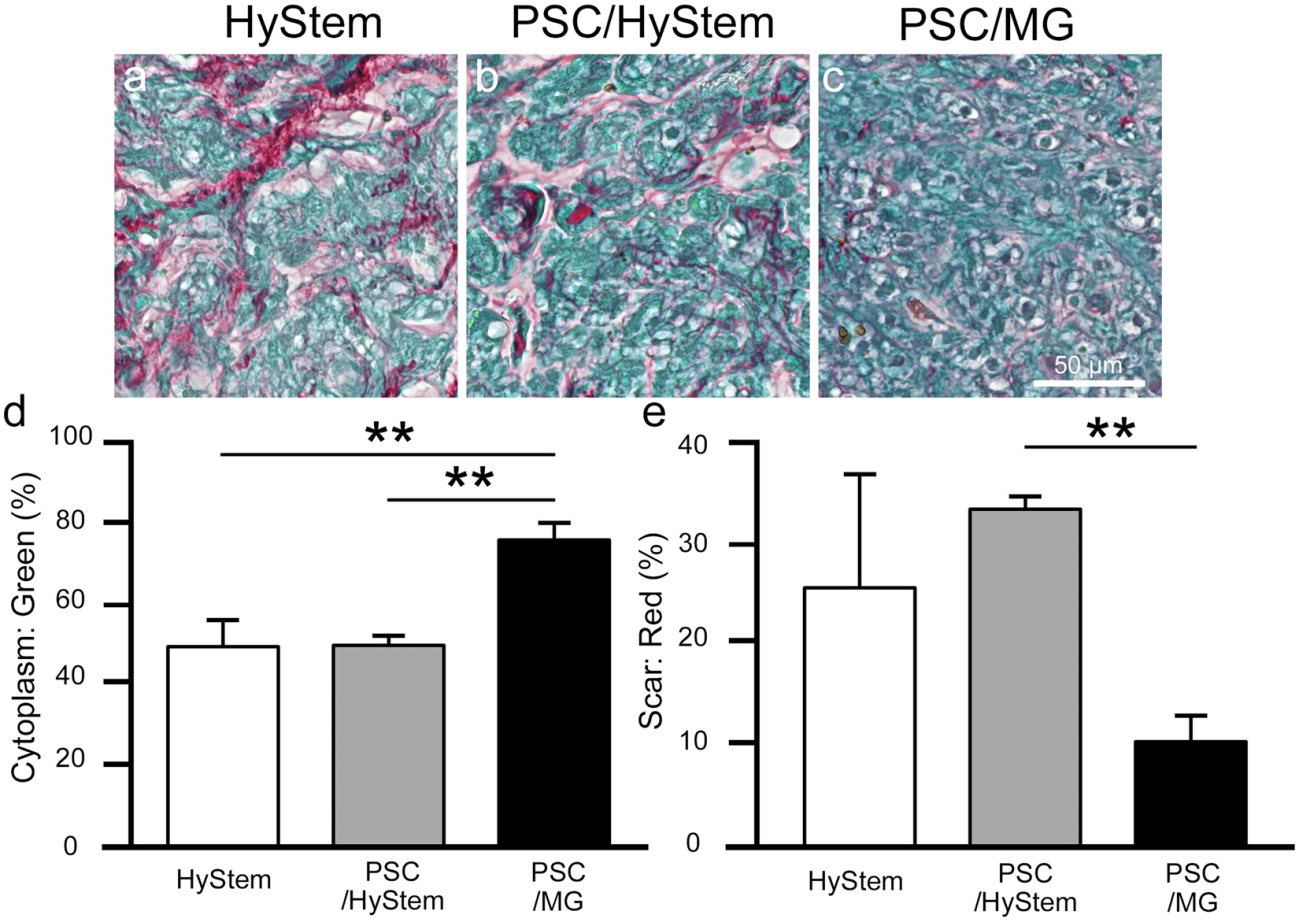
Relative size of the cytoplasm and collagen tissue in the graft area is shown. (**a**–**c**) High-power images of the graft area with Picro-sirius red staining. (**d**) Fraction of the cytoplasm area shown in green, **P < 0.01. (**e**) Fraction of the collagen tissue (scar) area, **P < 0.01.

**Figure 3:**
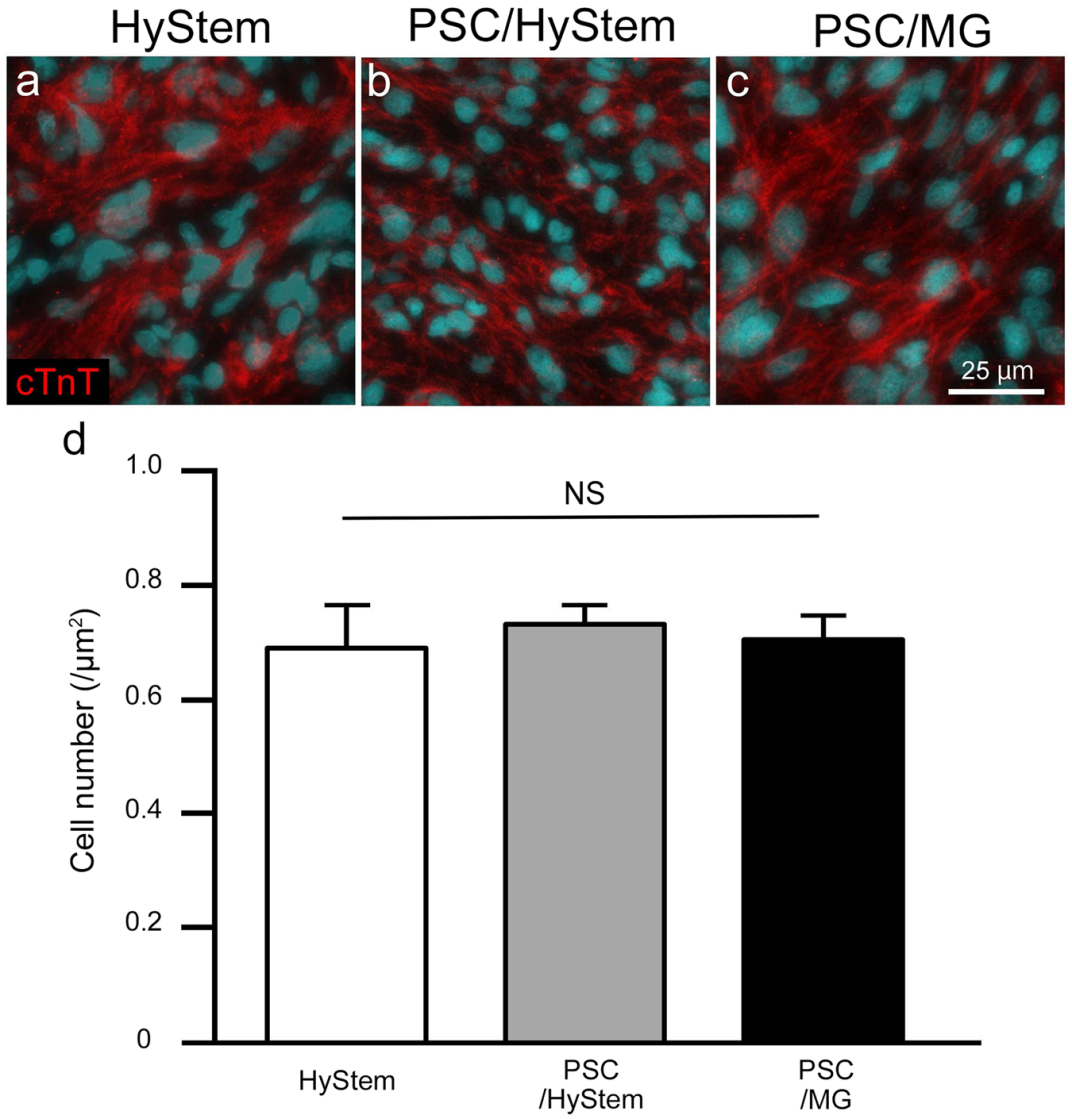
The number of grafted cardiomyocytes per unit area does not differ among the three experimental groups. (**a**–**c**) Representative images of the graft area stained against cardiac troponin T (cTnT). (**d**) The number of cells per unit area in the graft area.

**Figure 4:**
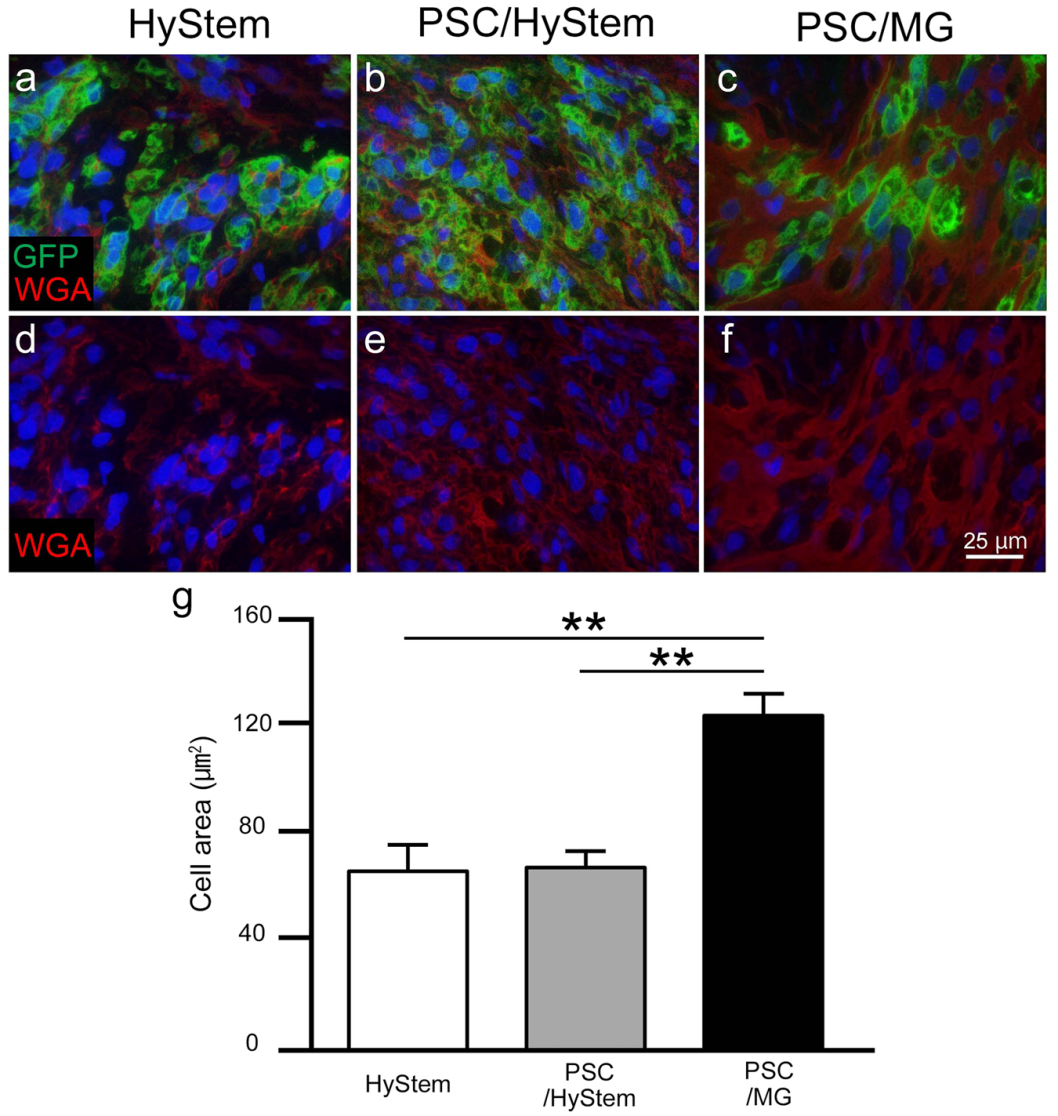
The PSC/MG group shows the largest graft cardiomyocytes in size. (**a**–**f**) Representative images of the graft area stained with GFP and wheat germ agglutinin. (**g**) Graft cell area, **P < 0.01. PSC, pro-survival cocktail; MG, Matrigel; GFP, green fluorescent protein.

Graft cells were histologically analysed. Picro-sirius red staining revealed less collagen tissue area in the grafts of the PSC/MG group compared to that in the other two groups (Figs 1d–f and 2a–c,e). Conversely, grafts in PSC/MG group had the largest cytoplasm area (Fig. 2d). These findings led to the speculation that grafts in the PSC/MG group contained a larger number of cells per unit area; therefore, we counted the number of cells in the grafts. Unexpectedly, the number of cells did not differ among the three experimental groups (Fig. 3). Next, the size of the graft cells was measured. The graft cells in the PSC/MG group showed larger cell area compared to those in the HyStem and PSC/HyStem groups (Fig. 4).

### Graft cardiomyocytes transplanted with PSC/MG showed a matured phenotype

**Figure 5:**
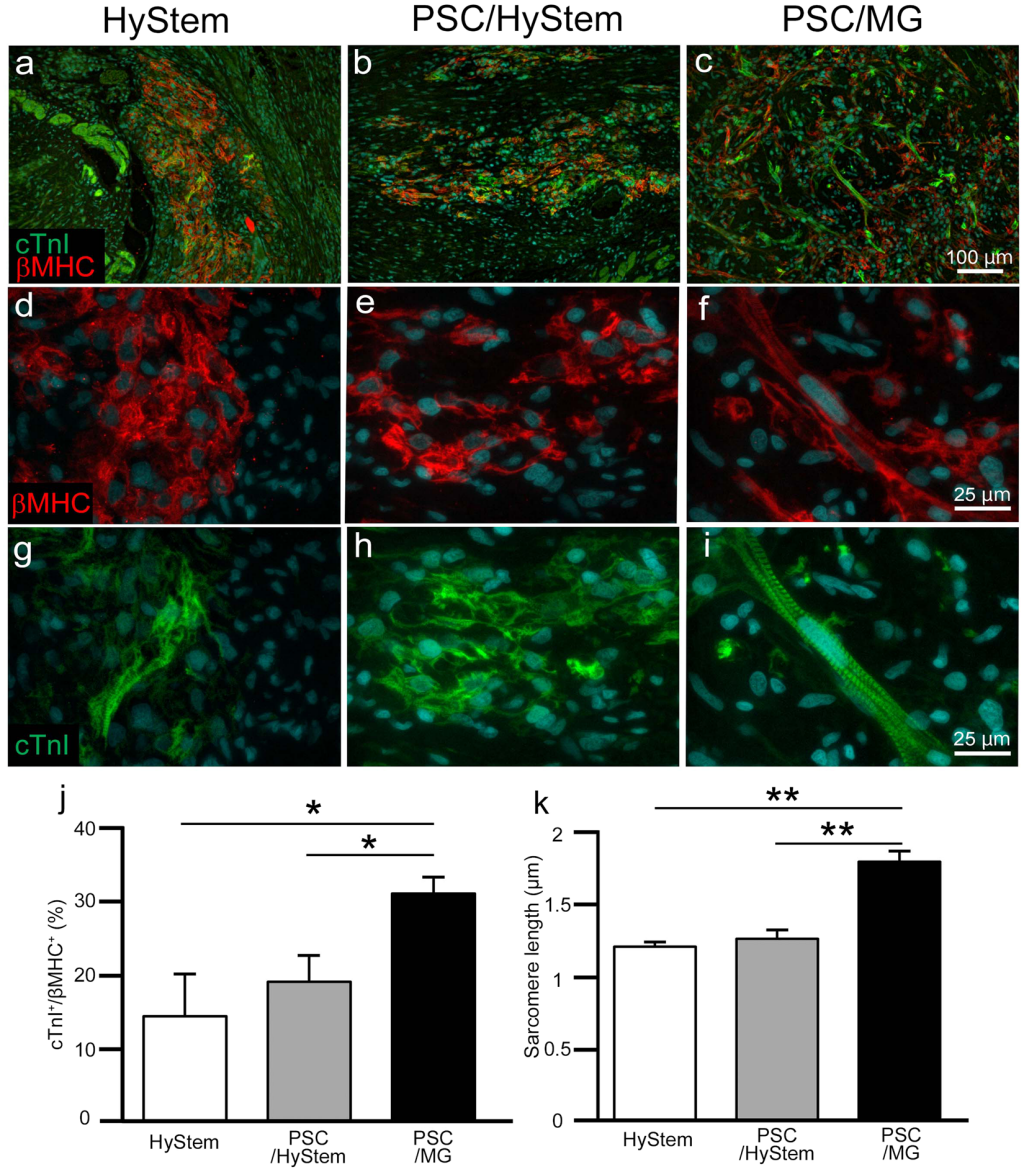
The maturation of grafted cardiomyocytes is shown. (**a**–**i**) Representative images of sections stained with cardiac troponin I (cTnI) and β-myosin heavy chain (βMHC). Note that the antibody against βMHC reacts with human-derived graft cardiomyocytes but not with rat-derived host cardiomyocytes. (**d**–**i**) Magnified images of (**a**–**c**). While the sarcomere structure was easily observed in the grafts of the PSC/MG group, it was relatively rarely observed in the HyStem and PSC/HyStem groups. (**j**) % of cTnI-positive cells in all grafted cardiomyocytes, *P < 0.05. (**k**) Length of the sarcomere in grafted cardiomyocytes, **P < 0.01. PSC, pro-survival cocktail; MG, Matrigel.

Matured cardiomyocytes are generally larger than immature cardiomyocytes^13^. The larger size of cardiomyocytes in the PSC/MG group was reminiscent of graft cardiomyocyte maturation; Matrigel has been shown to promote the maturation of cardiomyocytes^14^. Cardiac troponin I (cTnI) is a marker of matured cardiomyocytes^15^; thus, we counted the number of cTnI-positive graft cardiomyocytes. As expected, graft tissue in the PSC/MG group had significantly more cTnI-positive cardiomyocytes compared to that in the HyStem and PSC/HyStem groups (Fig. 5a–c,j). In addition, cardiomyocytes in the PSC/MG group showed a clear sarcomere structure; in contrast, sarcomere structure was rarely identified in the grafts of the HyStem and PSC/HyStem groups (Fig. 5d–i). We next measured the sarcomere length in graft cardiomyocytes; among the three groups, the PSC/MG group had the longest sarcomere length (Fig. 5g–i,k).

### Cardiac contractile function was not correlated with cardiac graft size

Several studies have shown that transplantation of human pluripotent stem cell-derived cardiomyocytes improves cardiac contractile function in acute or subacute myocardial infarction models^3–6^. Thus, we hypothesized that better engraftment of cardiomyocytes results in improved contractile function. To elucidate this hypothesis, we assessed cardiac contractile function using echocardiography before myocardial infarction induction (intact), a week after myocardial infarction induction (pre-transplantation), and four weeks after cell transplantation (post-transplantation) (Supplementary Fig. 2). Unexpectedly, transplantation of iPSC-CMs did not improve cardiac contraction; there were no group differences in left ventricular dimensions or fractional shortening (Supplementary Fig. 4). Accordingly, fractional shortening at 4 weeks post-transplantation did not correlate with the graft area (Supplementary Fig. 5).

## Discussion

The poor engraftment of cardiomyocytes in the infarcted heart has been attributed to several pathways, including ischemia^9^, inflammation^16^, and anoikis^17^. Pro-survival factors were designed to target all of these pathways^3^, and Matrigel was included to prevent anoikis. However, the use of Matrigel may not be clinically feasible. Hyaluronan and its major receptor, CD44S, have been shown to play an important role in the prevention of anoikis^18^. Indeed, the hyaluronan-based hydrogel, HyStem-C, has been shown to enhance cardiac graft survival in infarcted mouse hearts^11^.

However, we did not see a substantial amount of grafted cardiac tissue in the HyStem group in the present study. Parallel processes contribute to graft cell death and blocking one pathway can simply lead to cell death by another^3^. Accordingly, although a substantial amount of grafted tissue was observed in the PSC/HyStem and PSC/MG groups, the PSC/MG group had the largest grafted area (only the PSC/MG group had a significantly larger graft area compared to that in the HyStem group). In addition, graft cells in the PSC/MG group were larger in size, had a larger number of cTnI-positive cardiomyocytes, had a better-organized sarcomere structure, and had longer sarcomere length compared to those in the groups without Matrigel. These findings strongly suggest that grafted cardiomyocytes in the PSC/MG group were more mature compared to those in the PSC and PSC/HyStem groups. Consistent with this, a recent study showed that Matrigel promoted the maturation of iPS cell-derived cardiomyocytes^14^. Since an immature phenotype of pluripotent stem cell-derived cardiomyocytes is one of the remaining hurdles to overcome prior to clinical application^19^, future studies on graft survival enhancement should focus not only on graft size, but also the maturity of graft cardiomyocytes.

Although a previous study revealed that transplantation of human cardiomyocytes showed mechanical benefits in an injured rat heart^3^, cardiac contractile function did not correlate with graft size and maturity in the present study. While the mechanism by which transplanted human cardiomyocytes improves cardiac function in an injured rat heart is still obscure, the differences in study design, including the timing of cell transplantation and induction of myocardial infarction, may have contributed to this inconsistency in results. The genetically immune-deficient rat model is useful for assessing engraftment; however, larger animal models, in which the heart rate is better matched between host and graft animals, are superior for the evaluation of cardiac contractile function, as well as the electrical consequences. Indeed, when we transplanted cynomolgus iPS cell-derived cardiomyocytes into cynomolgus hearts, all of the graft cells were electrically coupled with host cardiomyocytes and cardiac contractile function was correlated with graft size^6^.

While almost all graft cells were solely composed of cardiomyocytes in our previous studies^4, 6, 20^, graft-derived cartilage tissue was observed in multiple recipients, despite a relatively higher purification of the transplanted cardiomyocytes. This may be related to protocol differences in the generation of cardiomyocytes. In addition, the subsequent purification process of cardiomyocytes might eliminate non-cardiac grafts; however, further transplantation studies are required to resolve this problem.

In conclusion, the pro-survival cocktail with Matrigel provided a larger graft and more mature iPS cell-derived cardiomyocytes compared to that for a hyaluronan-based pro-survival cocktail. Thus, hyaluronan does not appear to be a viable substitute for Matrigel. Further studies are required to establish a clinically feasible transplantation protocol.

## Methods

### Cell preparation

An undifferentiated iPS cell line, 253G1, expressing GCaMP3 was maintained using Essential 8 Medium (Thermo Fisher Scientific) without feeder cells. Cardiomyocytes were generated using a previously reported protocol. Briefly, iPS cell aggregates were allowed to attach to culture dishes in a cardiac differentiation medium (IMDM, Sigma-Aldrich) containing 1% MEM nonessential amino acid solution (Sigma-Aldrich), 1% penicillin-streptomycin (GIBCO), 2 mM L-glutamine (Sigma-Aldrich), 0.5 mM L-carnitine (Sigma-Aldrich), 0.001% 2-mercaptoethanol (GIBCO), 1% BSA (Wako), 4 mM CHIR (Axon), and 2 mM BIO (Calbiochem). On days 3–9, 10 mM KY02111 and/or other WNT inhibitors (XAV939) were added to the cell cultures, and the medium was changed every 2 days. Spontaneous beating was observed starting on days 10–14. After differentiation, cells were heat-shocked at 43 °C for 30 minutes on the day before harvesting, and cells were harvested and cryopreserved on days 40 ± 5. Cardiac purity was determined by using immunostaining of cardiac troponin T (clone 13-11) with flow cytometry. For the transplantation study, 2 × 10^7^ cells were thawed and diluted with 70 μL of either HyStem-C (Sigma-Aldrich) alone (HyStem), prepared according to the manufacturer’s instructions, five pro-surviving factors (100 mM ZVAD-FMK, 50 nM BCL-XL, 100 ng/mL IGF-1, 50 mM pinacidil, and 200 nM cyclosporine) with HyStem-C (PSC/HyStem), or the five factors with Matrigel (Corning, growth factor reduced; PSC/MG).

### Animal surgeries

Based on national regulations and guidelines, all experimental procedures were reviewed by the Committee for Animal Experiments and finally approved by the president of Shinshu University. T-cell deficient male F344/Njcl-rnu/rnu rats (aged 8–12 weeks, CLEA Japan) were used in the transplantation studies. Before thoracotomies, the animals were anesthetized via an intraperitoneal injection of 0.15 mg/kg medetomidine, 2 mg/kg midazolam, and 2.5 mg/kg butorphanol; and were intubated and mechanically ventilated with 1.5% isoflurane. After left intercostal thoracotomy, the left anterior descending artery was ligated with a 6-0 polypropylene suture (ETHICON) below the right atrial appendage level. On day 7 after the myocardial infarction, the animals underwent a second thoracotomy and the heart was exposed. iPSC-CMs (2 × 10^7^) diluted with HyStem, PSC/HyStem, or PSC/MG were delivered directly into the infarct and border zone via 2 injections using a 29-gauge injection needle. Subcutaneous Meloxicam was routinely administered to provide postoperative pain relief.

### Echocardiography

Echocardiography was performed before the induction of myocardial infarction (intact), a week after myocardial infarction (pre-transplantation), and 4 weeks after cell transplantation (post-transplantation). The animals were mechanically ventilated with 1.5% isoflurane, and the left-ventricular end-diastolic dimension (LVEDD), left-ventricular end-systolic dimension (LVESD), and heart rate were measured using transthoracic echocardiography (GE Vivid7) with a 10-MHz paediatric transducer. Fractional shortening (FS) was calculated using the following equation: FS = 100 × ((LVEDD− LVESD)/LVEDD). All measurements were taken over three consecutive cardiac cycles, which were averaged. An operator who was blinded to the study groups performed all measurements.

### Histology

Four weeks after cell transplantation, the animals were euthanized and the hearts were collected, sliced at 2-mm thickness using Slicer (Zivic), fixed with 4% paraformaldehyde, and imbedded with paraffin for histological analysis. All sections were routinely stained with haematoxylin and eosin (HE) and Picro-sirius red to determine the scar region. Immunohistochemistry was performed using primary antibodies against cardiac troponin T (clone 11–13), cardiac troponin I (rabbit polyclonal, Abcam), β myosin heavy chain (clone A4.951), and green fluorescent protein (GFP; rabbit polyclonal, Novus), followed by species-specific fluorescent (Molecular Probes) or biotin-conjugated (Vector Laboratories) secondary antibodies. For brightfield studies, chromogenic detection was performed with diaminobenzidine (DAB) followed by counterstaining with fast green or haematoxylin. Wheat germ agglutinin conjugated with Alexa Fluor 594 (Thermo Fisher) was used to determine cell boundaries.

### Quantification of histological sections

The number of cells in the graft area was quantified by counting the number of nuclei, rather than cytoplasm, as graft cardiomyocytes usually show inconspicuous cell membrane and alignment. Graft and scar areas were measured using NDP.view2 (Hamamatsu).

### Statistical analysis

Group differences in cell area and numbers, sarcomere length, and echocardiographic parameters were evaluated using analyses of variance (ANOVAs), and post-hoc comparisons were performed using Tukey’s multiple comparison tests. The relationship between graft area and fractional shortening was evaluated using the Pearson correlation. All values are expressed as mean ± standard error. All statistical analyses were performed using GraphPad Prism, and the threshold for significance was set at P < 0.05.

## Electronic supplementary material

Supplementary Information
