# Supplementary material for: Impact of extracellular matrix on engraftment and maturation of pluripotent stem cell-derived cardiomyocytes in a rat myocardial infarct model
Source: Sci Rep. 2017 Aug 17;7:8630. doi: 10.1038/s41598-017-09217-x (PMC5561148; doi:10.1038/s41598-017-09217-x)
Supplement: Supplementary file 1 — Supplementary Information [file 41598_2017_9217_MOESM1_ESM.pdf]

Impact of extracellular matrix on engraftment and maturation of pluripotent stem cell-derived cardiomyocytes in a rat myocardial infarct model

Tatsuki Ogasawara[1, 2, 3†], Satomi Okano[1, 2†], Hajime Ichimura[1, 2, 3], Shin Kadota[1, 2], Yuki Tanaka[1, 2, 3], Itsunari Minami[5], Motonari Uesugi[6], Yuko Wada[3], Naoto Saito[1], Kenji Okada[3], Koichiro Kuwahara[4], and Yuji Shiba[1, 2, 4]

[1]Institute for Biomedical Sciences,

Departments of Regenerative Science and Medicine[2], Cardiovascular Surgery[3], and Cardiovascular Medicine[4], Shinshu University, Matsumoto, Japan

[5]Department of Cell Design for Tissue Construction, Osaka University, Suita, Japan

[6]Institute for Integrated Cell-Material Sciences (WPI-iCeMS) and Institute for Chemical Research, Kyoto University, Uji, Japan.

[†]These authors contributed equally to this study.

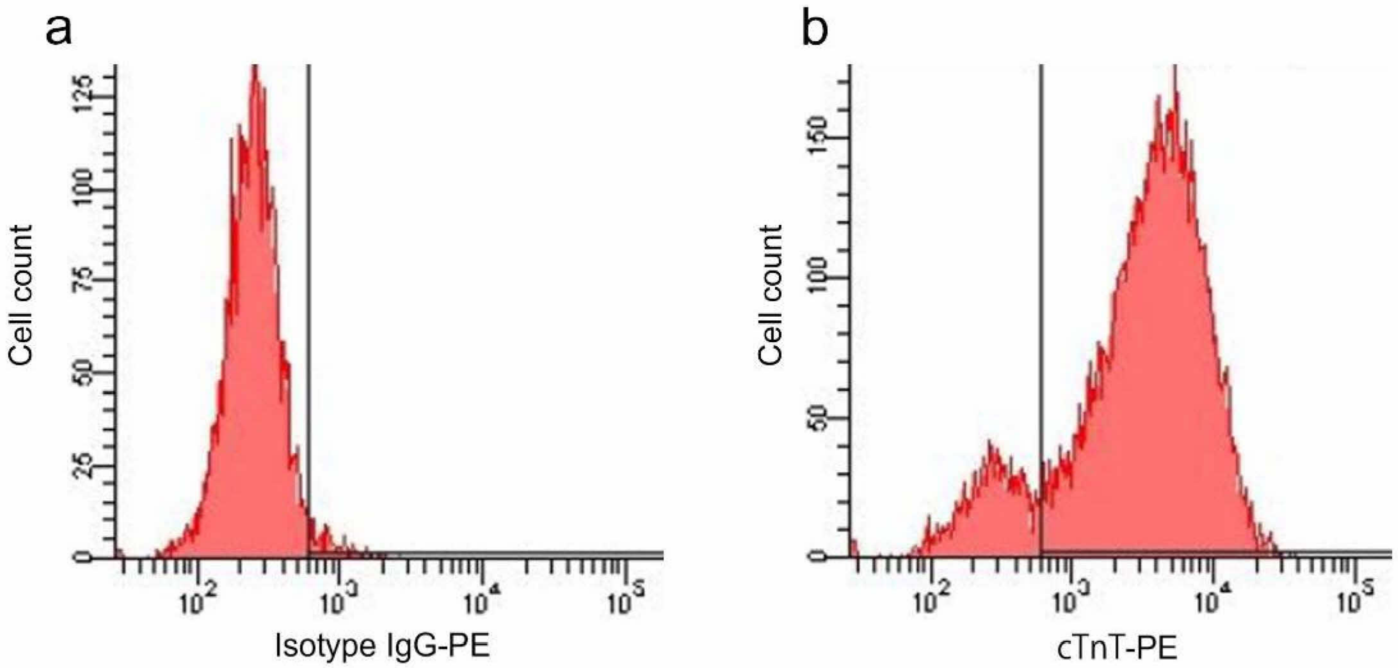

Supplementary Fig. 1 Flow cytometry for cardiomyocyte differentiation of human iPS cells.
Representative histograms of human iPS cell-derived cardiomyocytes after differentiation show 86.5% cTnT-expressing cells.

Supplementary Fig. 1

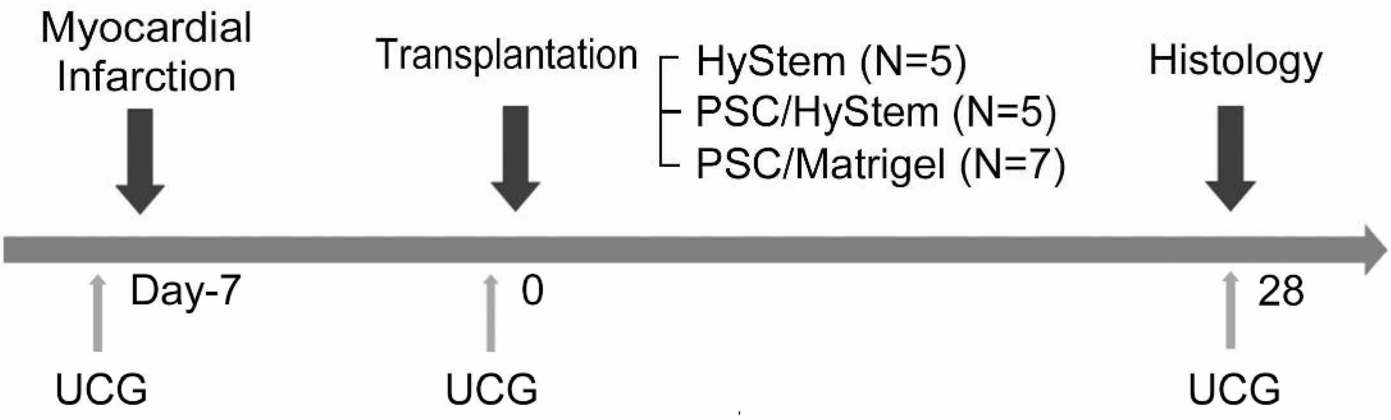

Supplementary Fig. 2 in vivo transplantation study protocol is depicted.
Male T cell-deficient rats underwent myocardial infarction induction by ligation of the left anterior descending artery. iPS cell-derived cardiomyocytes suspended with HyStem, PSC/HyStem, or PSC/MG were injected directly into the infarcted myocardium 7 days after myocardial infarction induction. Ultrasound cardiography (UCG) was performed on the day of myocardial infarction, cell transplantation, and 28 days after transplantation. All animals were euthanized on day 28 for histological analysis.
PSC, pro-survival cocktail; MG, Matrigel

Supplementary Fig. 2

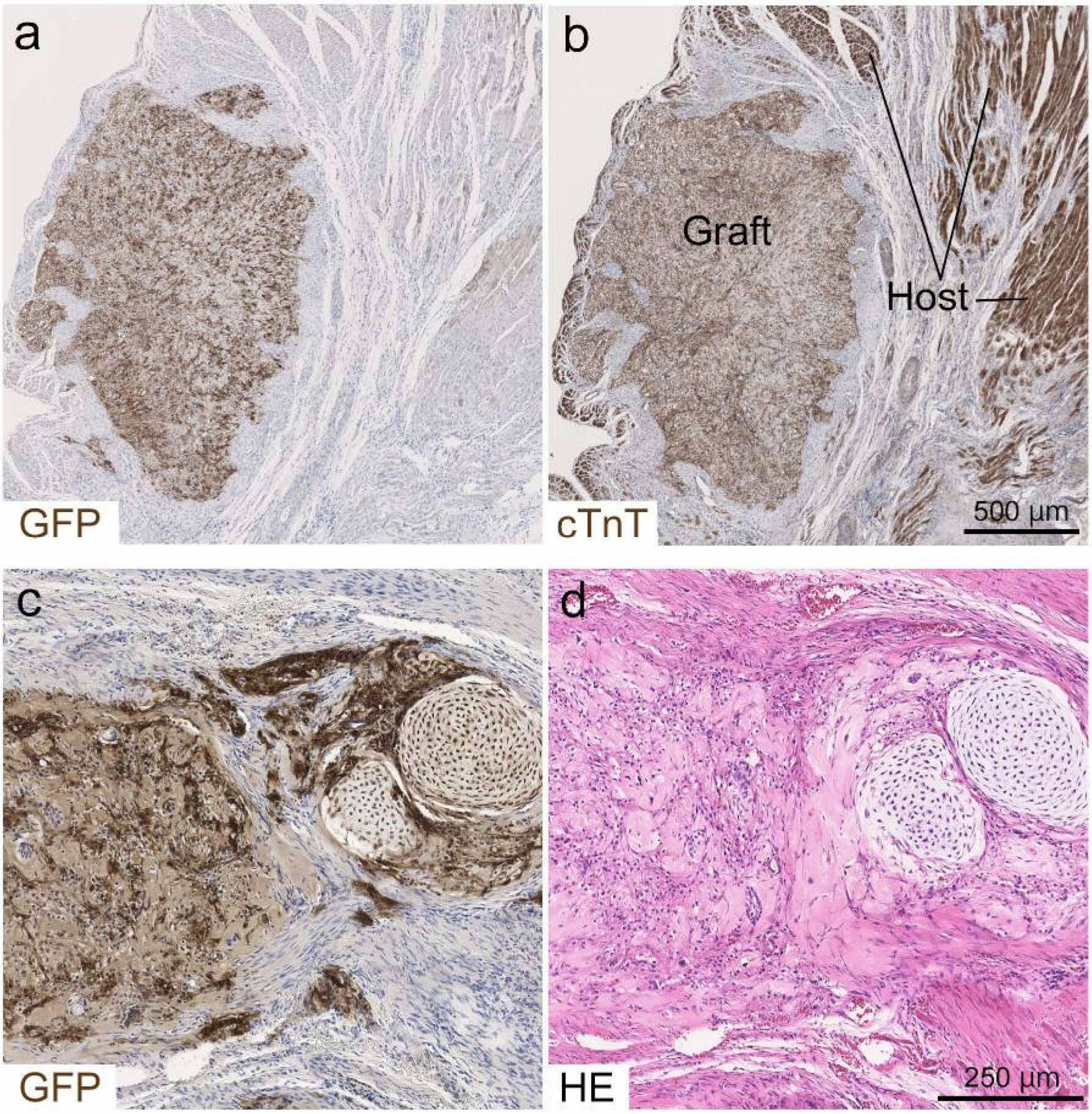

Supplementary Fig. 3 Histological analysis of graft tissue.
Most of the GFP+ graft area consisted of cardiac troponin T+ cardiomyocytes (**a, b**); however, a small portion of cartilage tissue was observed (**c, d**).
GFP, green fluorescent protein; cTnT, cardiac troponin T

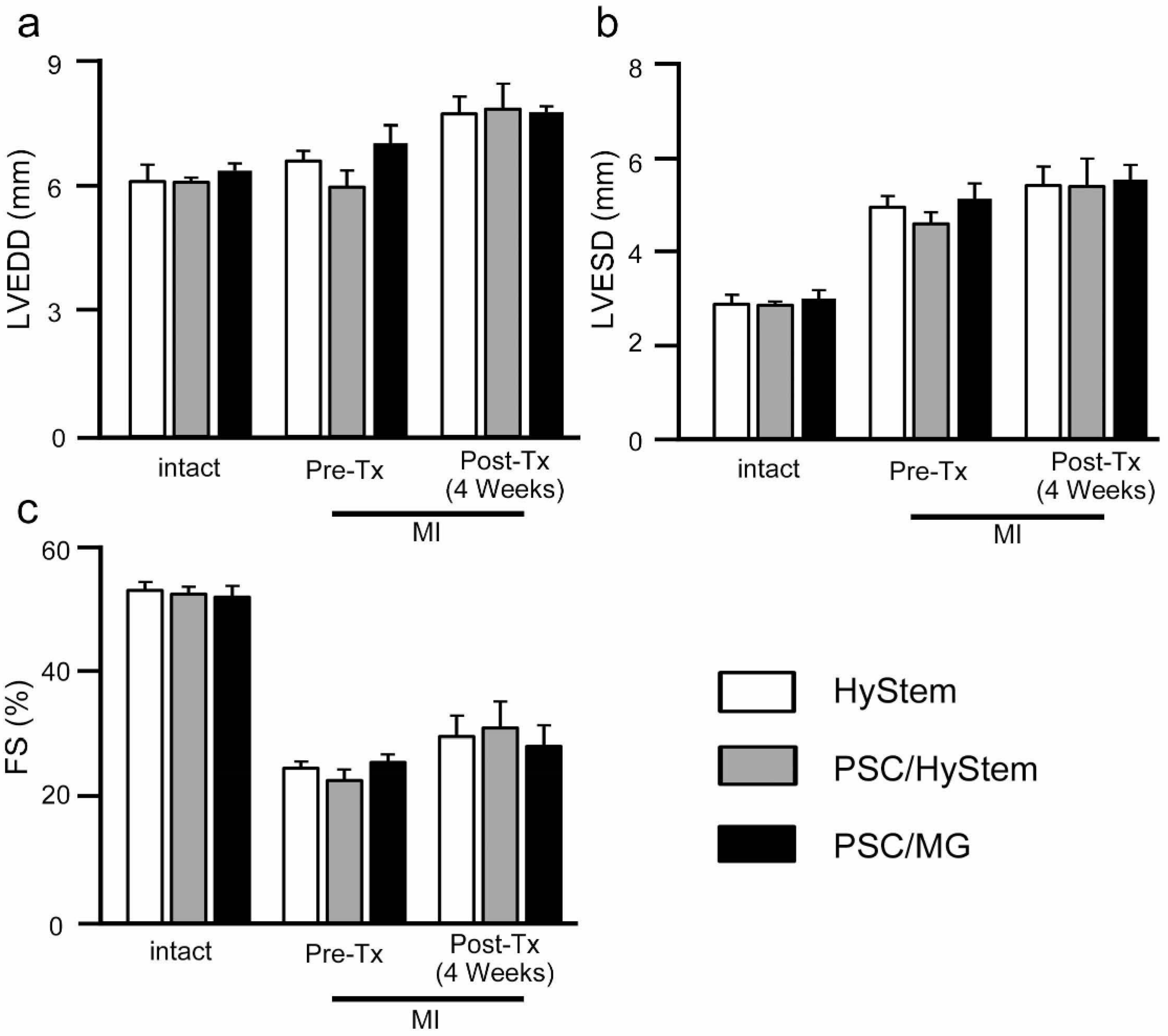

Supplementary Fig. 4 Ultrasound cardiographic assessment of left ventricular function is depicted.
Ultrasound cardiography (UCG) was performed before myocardial infarction (intact), before cell transplantation (Pre-Tx), and 4 weeks after cell transplantation (Post-Tx). Note that for all parameters, there were no significant differences among experimental groups and between Pre-Tx and Post-Tx.

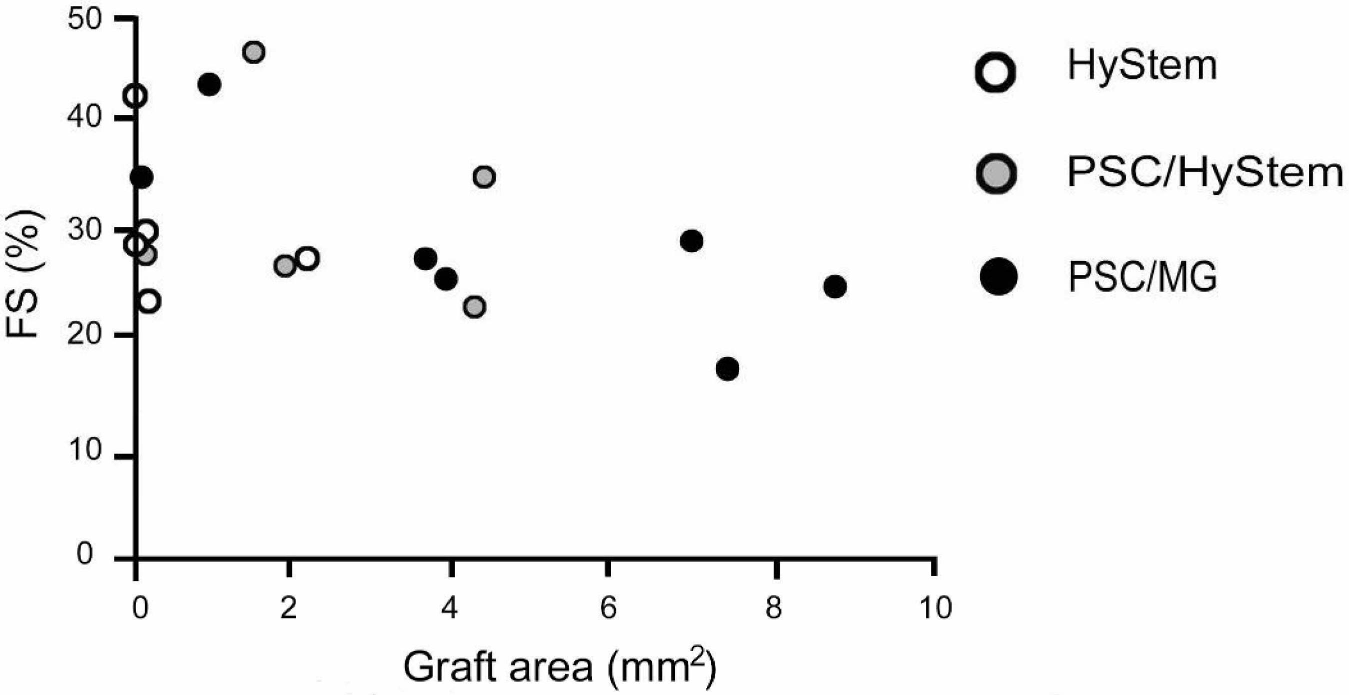

Supplementary Fig. 5 Relationship between left ventricular function and graft size is shown.
Dot-plot relationship between fractional shortening at 4 weeks post transplantation and histological graft area. The Pearson correlation (r=0.21) was not significant.

Supplementary figure legends

Supplementary Fig. 1 Flow cytometry for cardiomyocyte differentiation of human iPS cells.

Representative histograms of human iPS cell-derived cardiomyocytes after differentiation show 86.5% cTnT-expressing cells.

Supplementary Fig. 2 *in vivo* transplantation study protocol is depicted.

Male T cell-deficient rats underwent myocardial infarction induction by ligation of the left anterior descending artery. iPS cell-derived cardiomyocytes suspended with HyStem, PSC/HyStem, or PSC/MG were injected directly into the infarcted myocardium 7 days after myocardial infarction induction. Ultrasound cardiography (UCG) was performed on the day of myocardial infarction, cell transplantation, and 28 days after transplantation. All animals were euthanized on day 28 for histological analysis.

PSC, pro-survival cocktail; MG, Matrigel

Supplementary Fig. 3 Histological analysis of graft tissue.

Most of the GFP^+ graft area consisted of cardiac troponin T^+ cardiomyocytes (**a, b**); however, a

small portion of cartilage tissue was observed (**c, d**).

GFP, green fluorescent protein; cTnT, cardiac troponin T

Supplementary Fig. 4 Ultrasound cardiographic assessment of left ventricular function is depicted.

Ultrasound cardiography (UCG) was performed before myocardial infarction (intact), before cell transplantation (Pre-Tx), and 4 weeks after cell transplantation (Post-Tx). Note that for all parameters, there were no significant differences among experimental groups and between Pre-Tx and Post-Tx.

Supplementary Fig. 5 Relationship between left ventricular function and graft size is shown.

Dot-plot relationship between fractional shortening at 4 weeks post transplantation and histological graft area. The Pearson correlation (r=0.21) was not significant.
